# Automatic Counting of Microglial Cells in Healthy and Glaucomatous Mouse Retinas

**DOI:** 10.1371/journal.pone.0143278

**Published:** 2015-11-18

**Authors:** Pablo de Gracia, Beatriz I. Gallego, Blanca Rojas, Ana I. Ramírez, Rosa de Hoz, Juan J. Salazar, Alberto Triviño, José M. Ramírez

**Affiliations:** 1 Department of Neurobiology, Barrow Neurological Institute, St. Joseph’s Hospital and Medical Center, Phoenix, Arizona, United States of America; 2 Instituto de Investigaciones Oftalmológicas Ramón Castroviejo, Universidad Complutense de Madrid, Madrid, Spain; 3 Facultad de Óptica y Optometría, Universidad Complutense de Madrid, Madrid, Spain; 4 Facultad de Medicina, Universidad Complutense de Madrid, Madrid, Spain; Universidade Federal do ABC, BRAZIL

## Abstract

Proliferation of microglial cells has been considered a sign of glial activation and a hallmark of ongoing neurodegenerative diseases. Microglia activation is analyzed in animal models of different eye diseases. Numerous retinal samples are required for each of these studies to obtain relevant data of statistical significance. Because manual quantification of microglial cells is time consuming, the aim of this study was develop an algorithm for automatic identification of retinal microglia. Two groups of adult male Swiss mice were used: age-matched controls (naïve, n = 6) and mice subjected to unilateral laser-induced ocular hypertension (lasered; n = 9). In the latter group, both hypertensive eyes and contralateral untreated retinas were analyzed. Retinal whole mounts were immunostained with anti Iba-1 for detecting microglial cell populations. A new algorithm was developed in MATLAB for microglial quantification; it enabled the quantification of microglial cells in the inner and outer plexiform layers and evaluates the area of the retina occupied by Iba-1+ microglia in the nerve fiber-ganglion cell layer. The automatic method was applied to a set of 6,000 images. To validate the algorithm, mouse retinas were evaluated both manually and computationally; the program correctly assessed the number of cells (Pearson correlation R = 0.94 and R = 0.98 for the inner and outer plexiform layers respectively). Statistically significant differences in glial cell number were found between naïve, lasered eyes and contralateral eyes (P<0.05, naïve versus contralateral eyes; P<0.001, naïve versus lasered eyes and contralateral versus lasered eyes). The algorithm developed is a reliable and fast tool that can evaluate the number of microglial cells in naïve mouse retinas and in retinas exhibiting proliferation. The implementation of this new automatic method can enable faster quantification of microglial cells in retinal pathologies.

## Introduction

Microglial cells are the primary immune-responsive cells in the central nervous system. They serve in the surveillance, maintenance, protection, and restoration of nervous system homeostasis. They are distributed in the parenchyma of the brain, the spinal cord, and also the retina [1]. Although microglial cells are involved in vital tasks for the survival of neurons [2], microglia have also been implicated as a causative factor in a range of neurodegenerative disorders[3–6]. Under stress conditions that might put neuronal survival at risk, microglial cells are reactivated and become capable of undergoing proliferative processes and interactions with damaged cells[7,8].

Among the ocular neurodegenerative diseases, glaucoma constitutes the second most frequent cause of irreversible blindness in first-world countries [9]. Glaucoma’s pathology is a chronic, multifactorial optic neuropathy, characterized by the damage of the axons of the retinal ganglion cells, which ultimately results in the death of these neurons [10,11]. Ocular hypertension (OHT) is a major risk factor for developing glaucoma; however, the exact mechanisms implicated in its physiopathology are still unknown. It has been reported that microglial cells play an important role in development of glaucoma [12]. Microglial proliferation, among other activation features, has been reported in glaucoma in both human [13] and experimental animal models [13–22]. Recently, in a mouse model of unilateral laser-induced OHT, microglial proliferation occurred not only in OHT eyes but also in the contralateral eyes [23,24]. Therefore the quantification of microglial cells provides information about ongoing stress situations in the nervous system, including the retina.

Quantitative microglial studies often require thousands of images of numerous specimens to provide statistically significant results. These studies usually involve comparisons between normal and damaged specimens. In addition, in the retina, microglial cells are distributed in several layers—photoreceptor layer (PRL), outer plexiform layer (OPL), inner plexiform layer (IPL), nerve fiber layer (NFL), and ganglion cell layer (GCL)—resulting in a large number of images per retina to be analyzed. Manual microglial counting methods are time consuming and tedious. Previously, different computational approaches to develop custom algorithms for counting different types of nervous system cells have been developed [25–31]. Free software packages (e.g., ImageJ open-source software) also offer tools to identify structures of generally symmetric shapes. Nevertheless, retinal microglial cells exhibit a heterogeneous and complex morphology. In addition, the activation of microglial cells induces changes in their cellular morphology, making the task of identifying microglial cells across different conditions difficult.

Here we present a new algorithm that allows automatic counting of retinal microglial cells in mice samples. The algorithm can be applied to retinal microglial cells from naïve animals and in both the OPL and IPL from eyes showing proliferative responses in a mouse model of unilateral laser-inducer OHT. This method also allows us to evaluate the area of the retina occupied by microglial cells in the combined NFL-GCL.

## Material and Methods

### Ethics Statement

Mice were treated in accordance with the Spanish Laws and the Guidelines for Humane Endpoints for Animals Used in Biomedical Research. This study was approved by the Ethics Committee for Animal Research of the University Complutense of Madrid. Also, animal treatment followed institutional guidelines, European Union regulations for the use of animals in research, and the Association for Research in Vision and Ophthalmology (ARVO) statement for the use of animals in ophthalmic and vision research.

### Animal Model and Sample Preparation: Immunohistochemistry

The experiments were performed on 12-week-old (weight, 40–450 g) adult male albino Swiss mice. Two groups were considered for study: an age-matched control (naïve, n = 6) and a lasered group (n = 9), with analyses conducted on both OHT eyes and contralateral eyes in the latter group. The induction of OHT and measurement of intraocular pressure were performed according to previously published protocols [23,24,32,33]. In brief, left eyes were treated in a single session with a series of diode laser burns (532 nm) aimed at the limbal and episcleral veins.

With the mice under deep anesthesia (injection of 75-mg/kg Ketamine and 10-mg/kg Xylazine), the intraocular pressure (IOP) was measured in both eyes with a rebound tonometer (Tono-Lab, Tiolat, Helsinki, Finland) [32] prior to and 24 hours, 48 hours, and 1 week after laser treatment for the lasered group, and before being killed for the naïve group. At each time point, six consecutive readings were taken for each eye and averaged. To avoid fluctuations of the IOP due to the circadian rhythm in albino Swiss mice [34], or due to the rise of the IOP itself [35], we tested the IOP consistently around the same time, preferentially in the morning and directly after deep anesthesia in all animals (lasered group and naïve). Animals were killed 2 weeks after lasering with an intraperitoneal overdose of pentobarbital.

Data for the statistical analysis of IOP values were introduced and processed in SPSS 19.0 (IBM, Armonk, NY). Data are shown as mean ± SD. Statistical analyses were performed with analysis of variance (ANOVA) and Bonferroni tests to identify differences among IOP values of the OHT, contralateral, and naïve eyes.

To analyze microglial cell populations in the mice, retinas from naïve, OHT eyes, and contralateral eyes were dissected and processed as retinal whole mounts. Samples were immunostained as described elsewhere [23,24,33] with the antibodies rabbit anti Iba-1 (Wako, Osaka, Japan) in a 1/500 dilution (primary antibody) and donkey anti-rabbit Alexa Fluor 594 (Invitrogen, Paisley, UK) diluted 1/800 (secondary antibody).

### Retinal Image Analysis

Retinas were analyzed with an ApoTome device (Zeiss, Germany) and photographed with a digital high-resolution camera (Cool SNAP Photometrics, USA) both coupled to a fluorescence microscope (Zeiss Axioplan 2 Imaging Microscope, Germany) equipped with an appropriate filter for fluorescence emission spectra of Alexa fluor 594 (Zeiss Filter set 64, Germany). The ApoTome uses the “structured-illumination” method that enables conventional microscopy to create optical sections through the specimen and thereby improve the contrast and resolution along the optical axis. Z stacks acquired were analyzed in Axiovision version 4.2 (Zeiss, Germany).

**Fig 1:**
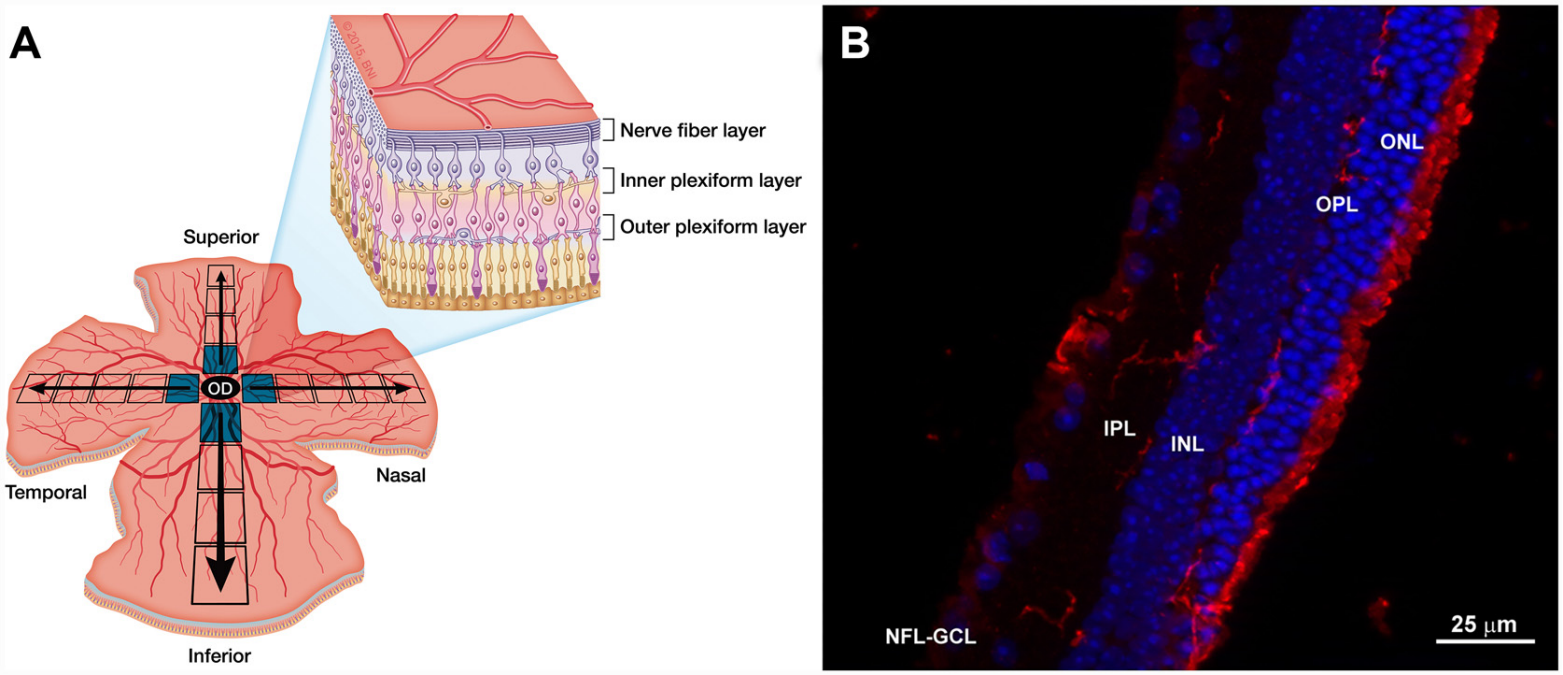
Retinal analysis. (A) Illustration of retinal whole mount showing areas of retina selected for quantitative analysis of Iba-1+ cells toward the horizontal and vertical meridian. (B) Retinal section with Iba-1 immunostaining. Iba-1+ cells (in red) are distributed in different retinal layers. (INL: inner nuclear layer; IPL: inner plexiform layer; NFL-GCL: nerve fiber layer-ganglion cell layer; ONL: outer nuclear layer; OPL: outer plexiform layer). *Used with permission from Barrow Neurological Institute*, *Phoenix*, *Arizona*.

For quantitative analysis of the Iba-1+ cells, equivalent areas of the retina were consistently selected and photographed for each retinal whole mount in both the vertical and horizontal meridians that cross the optic nerve (Fig 1). All subsequent fields analyzed were contiguous to ensure that no portion of the retinal whole-mount would be omitted or duplicated. Each meridian was analyzed using the motorized stage of the microscope to scan its whole extension along the X-Y axis, giving an approximate total of 550 fields evaluated. Photographs were taken at 20× magnification, systematically yielding an area of 0.1502 mm^2^. Additionally, to quantify the Iba-1+ cells in the OPL, IPL, and NFL-GCL, we analyzed the whole preparation along the Z axis in depth every 2 μm. The means of images in every z stack in the OPL were 6.78±0.35 for control eyes, 4.87±0.57 for contralateral eyes, and 6.49±1.43 for OHT eyes, and the means in the IPL were 8.77±0.82 for control eyes, 7.39±1.79 for contralateral eyes, and 5.43±1.60 for OHT eyes.

### Algorithm Development

A new algorithm consisting of segmentation, thresholding, and control of distances was implemented in MATLAB (MathWorks, Natick, Massachusetts, USA) and used to obtain the number of microglial cells and their position in the IPL and the OPL and to calculate the area of the retina occupied by microglial cells in the NFL-GCL.

**Fig 2:**
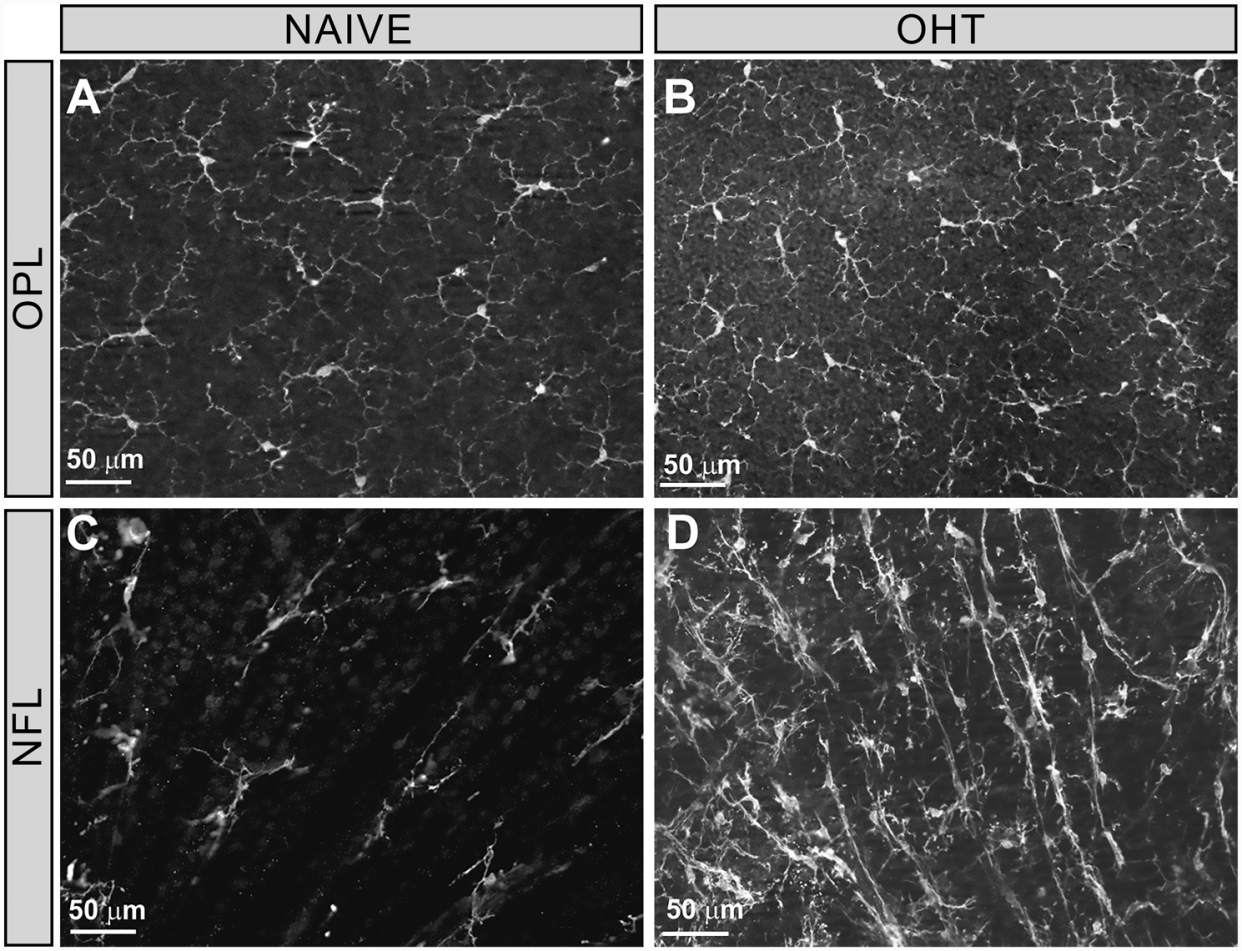
Iba-1+ microglial distribution in the retina. Retinal whole mount with Iba-1 immunostaining. (A-B) Iba-1+ microglias formed a cellular net in the outer plexiform layers without overlap between them, allowing their individual identification. (C-D) In the NFL-GCL, the distribution of Iba-1+ microglial cells makes it difficult to distinguish one from another. (NFL: nerve fiber layer; OHT, ocular hypertension; OPL: outer plexiform layer). *Used with permission from Barrow Neurological Institute*, *Phoenix*, *Arizona*.

In the IPL and OPL, microglial cells were distributed throughout the retina in a mosaic-like fashion without overlap between neighboring cells (Fig 2A and 2B). This feature allows the algorithms to automatically determine the number of microglial cells. By contrast, in the NFL-GCL, cell separation and distribution did not fulfill criteria for automatic individual cell counting in naïve retinas (Fig 2C), and was even more difficult in OHT retinas (Fig 2D) where proliferative processes could be observed [23,24]. Therefore, we quantified the area of the retina occupied by Iba-1+ cells in the NFL-GCL.

**Fig 3:**
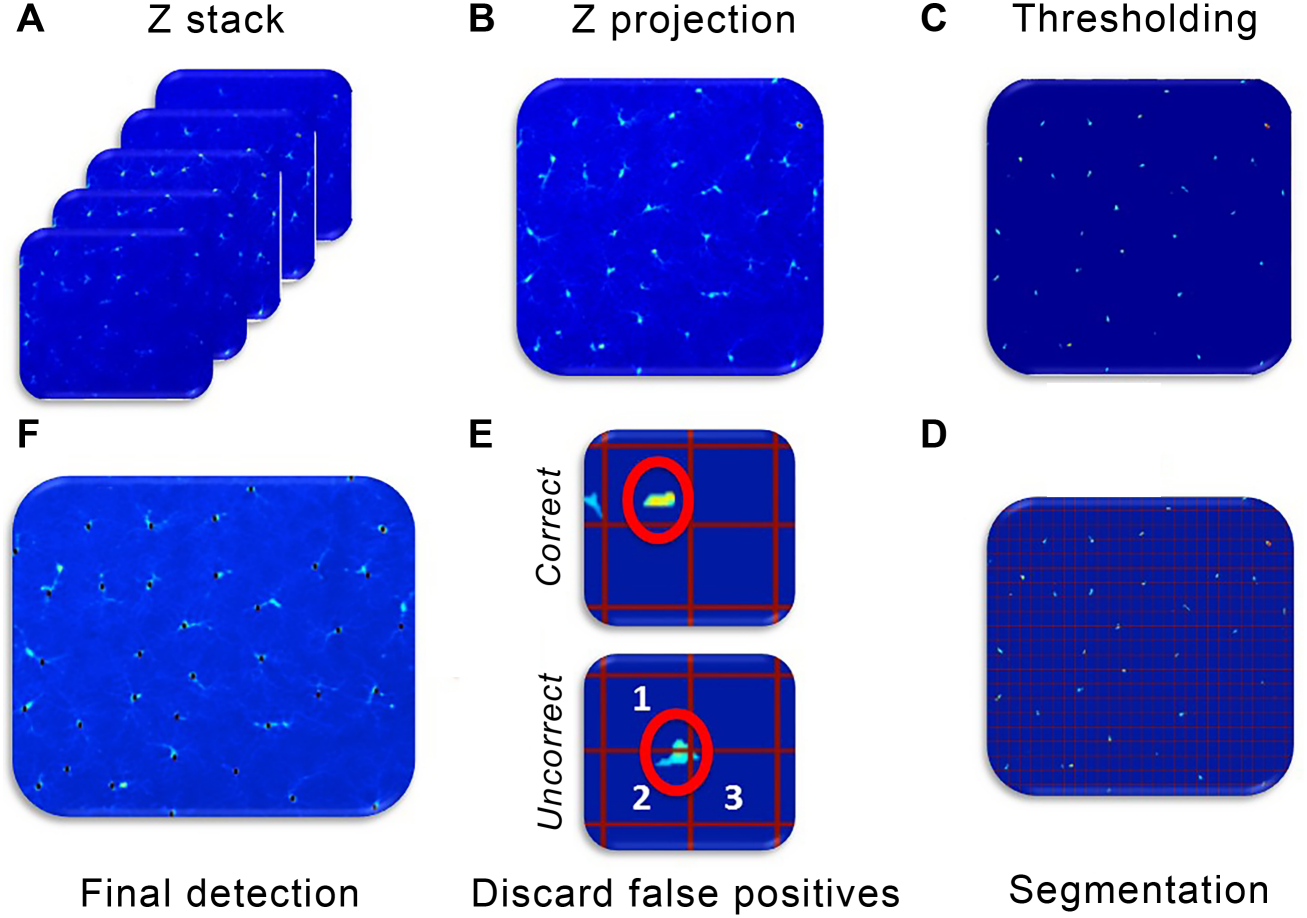
Work flow describing the algorithm for Iba-1+ microglial cell counting. (A) Sets of images are taken by scanning a layer of the retina every 2 μm in depth (Z direction). The averaged image of the set of images taken in depth is the Z projection (B); afterward, image thresholding (C) and segmentation (D) into 50×50-pixel regions of interest are performed. Examples of correct cell identification and multiple identification of the same cell in various segments (E) and final detection of cells (F) are shown. *Used with permission from Barrow Neurological Institute*, *Phoenix*, *Arizona*.

A scheme was developed for the process of counting cells in the IPL and the OPL (Fig 3). The algorithm started by averaging the images in depth taken for the same area and layer (Fig 3A). The result of this operation is referred as the *Z projection* of the area and layer under study (Fig 3B). Two operations are performed with this image. First, to create the *threshold*, the image was normalized to the pixel with the maximum value in the image, after which the image values are within a range from 0 to 1. Second, a threshold operation was performed that set all the values under the normalized image <0.2 to 0 and preserved the rest (Fig 3C). The resulting image is *segmented* in 50×50-pixel squares. This size was selected because even the smallest microglial cells occupied at least 4 different sectors (Fig 3D). Next, the center of mass of each 50×50 segment was calculated. If all the values within a box were 0, no result was obtained from that box. Therefore, a rough estimate of the number of cells was obtained by totaling the number of boxes in which the calculation of the center of mass was different from 0 at this stage. Although no box was expected to get signal from more than 1 cell, 2 boxes could have signal from the same cell. Therefore, we discarded the false positives by introducing a condition of minimum distance between 2 different cells (Fig 3E). All the points that were within that minimum distance were considered to belong to the same cell and only 1 of the points was counted as a cell. Together, these operations created the final detection of cells (Fig 3F).

**Fig 4:**
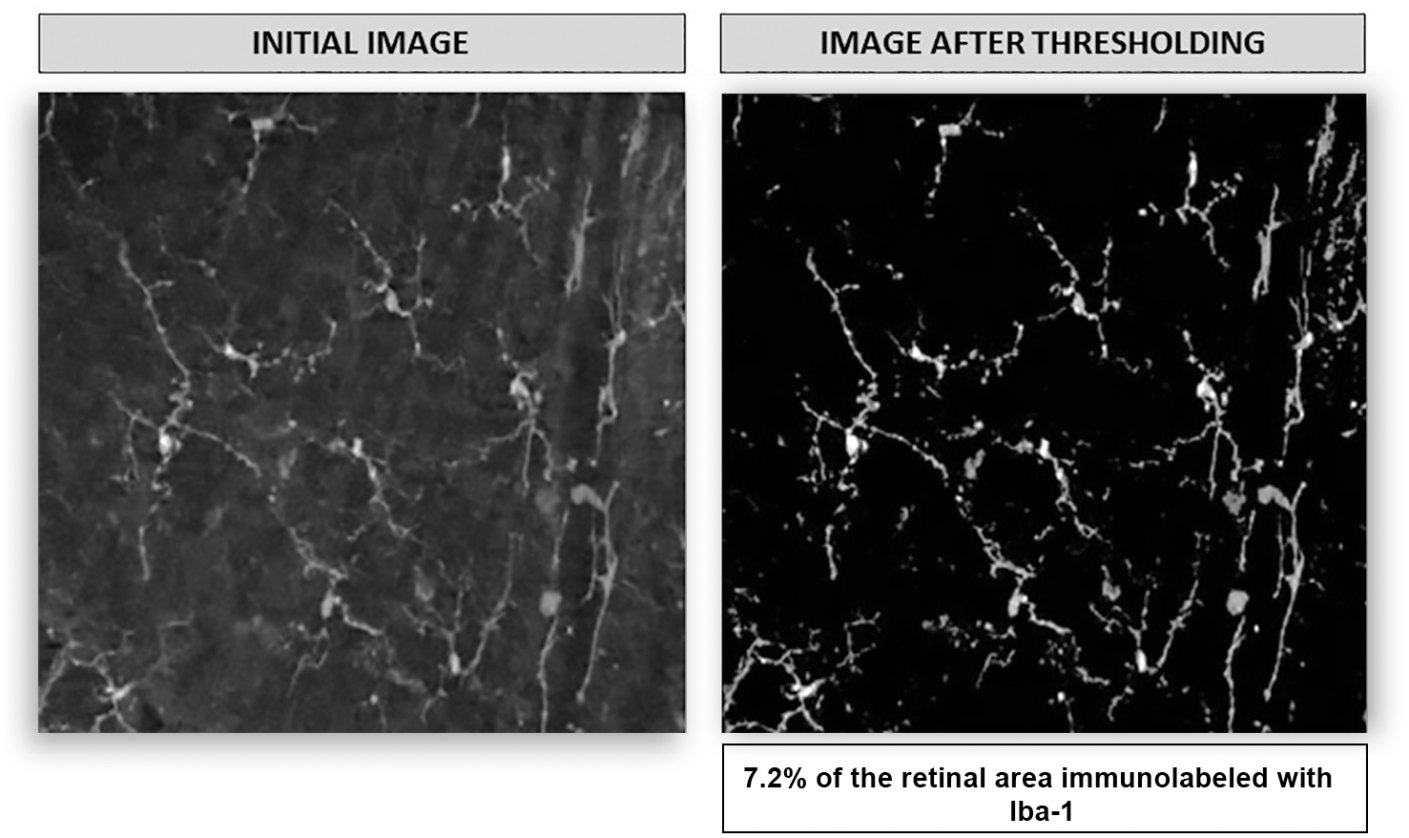
Retinal area labeled with Iba-1 in the NFL-GCL. Left panel shows a microphotograph from the NFL-GCL. Right panel shows the same image after thresholding. The percentage of retinal area labeled with Iba-1 in this example is 7.2%. *Used with permission from Barrow Neurological Institute*, *Phoenix*, *Arizona*.

To analyze the area of the retina labeled with Iba-1 in the NFL-GCL, a binarized image was computed after the threshold operation by assigning a value of 1 to all the values different from 0. Then all the pixels of the image were added and the result was divided by the total number of pixels (image size 1380×1040 pixels) to obtain the percentage of the image labeled with Iba-1 (Fig 4).

**Fig 5:**
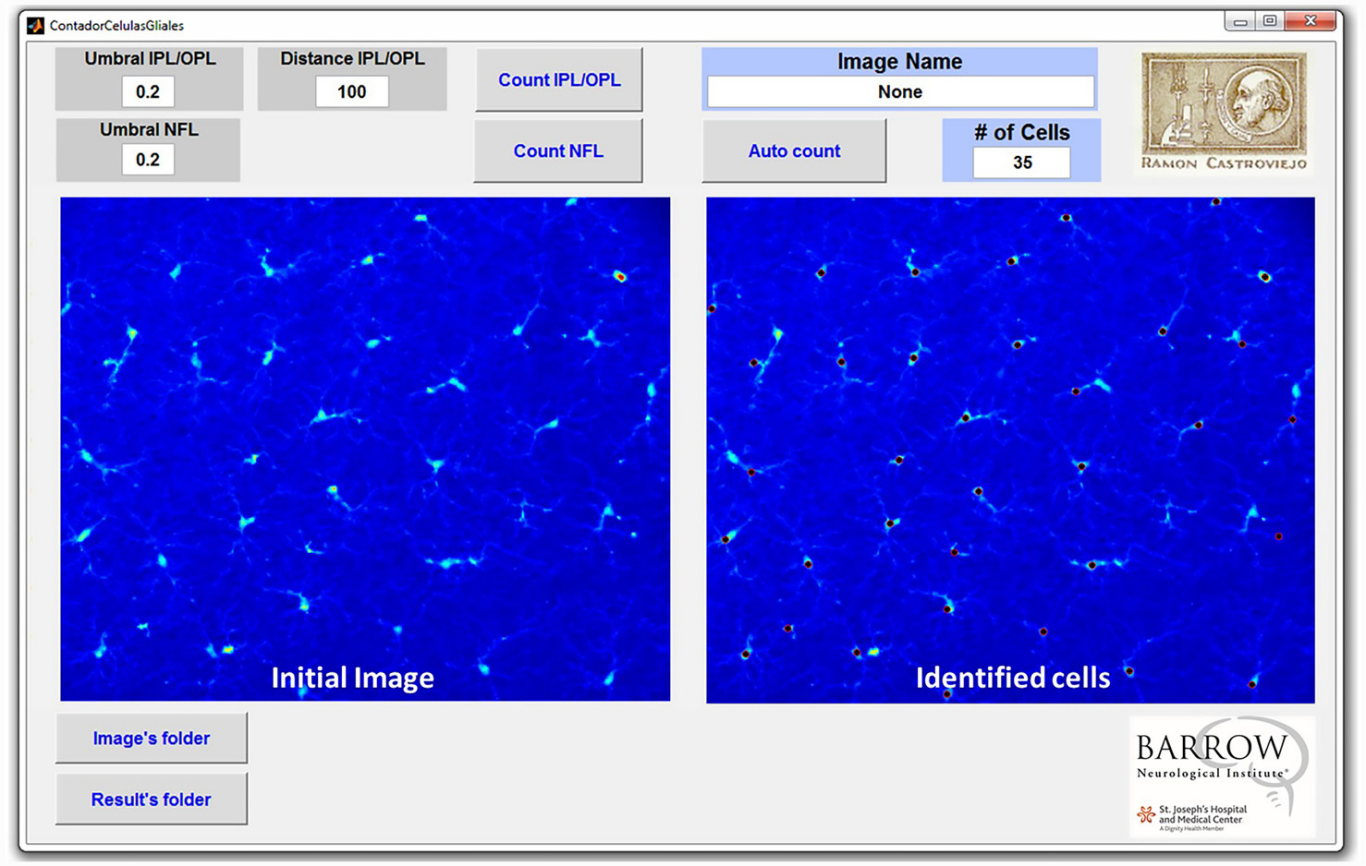
Image of the program interface. The umbral inner plexiform layer/outer plexiform layer (IPL/OPL) and distance IPL/OPL are used to introduce the values required to evaluate the number of cells in the IPL and OPL of the retina. The umbral value determines the threshold of intensity on the image over which a pixel is considered as part of a microglial cell. Distance represents the minimum distance under which two positive values found in different sectors are considered to belong to the same cell (it is used to discard false positives). The umbral nerve fiber layer (NFL) is used to introduce the umbral value to evaluate the retinal area immunolabeled with Iba-1 in the NFL-ganglion cell layer (GCL). “Count IPL/OPL” counts the number of cells in one image of the IPL/OPL layer. The “Count NFL” evaluates the percentage of retinal area immunolabeled with Iba-1 in the NFL. “Autocount” provides the number of cells present in the IPL/OPL layers and the percentage of retinal area immunolabeled with Iba-1 in the NFL of all the images found in the selected “Images folder,” and produces an Excel file with the results in the “Results folder” selected. When pressing “Autocount” the name of the image currently measured is presented under “Image Name.” The image shown in this figure is the result of pressing the “Count IPL/OPL” button. *Used with permission from Barrow Neurological Institute*, *Phoenix*, *Arizona*.

In addition, an interface for the algorithm was developed to facilitate the use of the program; an example and explanation of its functions can be seen in Fig 5.

### Manual Counts

A subset of 20 fields per layer (OPL and IPL) in naïve, contralateral eyes, and OHT eyes were chosen randomly, totaling 120 fields analyzed. Microglias in the IPL and OPL in mice retina are distributed in such a way that each Iba-1+ labeled microglia can be easily distinguished from others, allowing cell counting. We quantified Iba-1+ microglial somas in each selected photograph of the retinal whole mount using the Axiovision (Zeiss, Germany) manual counting tool.

### Statistical Analysis

Data for the statistical analyses were processed in SPSS 22.0 (IBM). Data are shown as mean ± standard deviation (SD). Statistical analyses were performed with Wilcoxon signed rank test to identify differences between automatic and manual quantitative analyses. Cell counts obtained by the automated method were compared with those obtained by the manual method using the Pearson correlation test. Differences were considered significant when P was <0.05.

## Results

To validate the new algorithm, the results of the automatic counting of cells without any further human interaction for the IPL and the OPL were compared with a direct manual quantification of microglial cells by an investigator.

The IOP values of OHT-eyes (29.6±4.4 mmHg) significantly differed from naïve values (16.2±3.1 mmHg; *P* < 0.001, ANOVA with Bonferroni) and contralateral eyes (15.4±1.6 mmHg; *P* < 0.001, ANOVA with Bonferroni). No significant differences were found between contralateral and naïve eyes.

**Fig 6:**
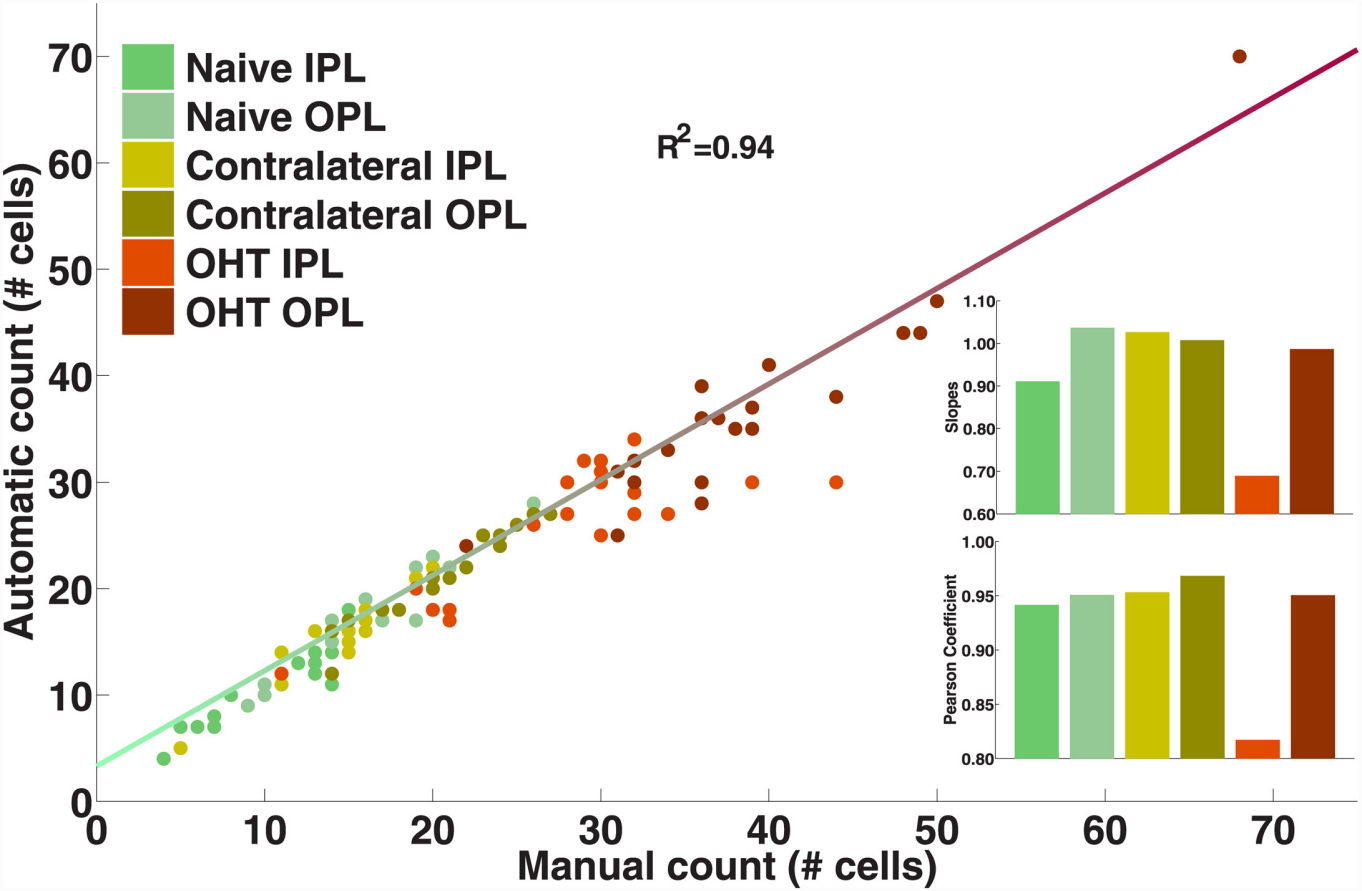
Number of microglial cells counted with the automatic method as a function of the direct human observation of the samples. Insets represent the slopes (top) and the Pearson coefficient (bottom) for each subgroup. (IPL: inner plexiform layer; OHT: ocular hypertension; OPL: outer plexiform layer). *Used with permission from Barrow Neurological Institute*, *Phoenix*, *Arizona*.

Fig 6 shows the number of cells detected in each retina from the study in the IPL and the OPL by the automatic algorithm as a function of those obtained by direct human quantification of microglial cells. Of the variability found in the automatic counting, 94% is explained by the count produced by direct observation of the samples. The slopes of the linear fitting (0.94±0.06) and the Pearson coefficients (0.93±0.02) for each subset of the sample are shown as insets.

**Fig 7:**
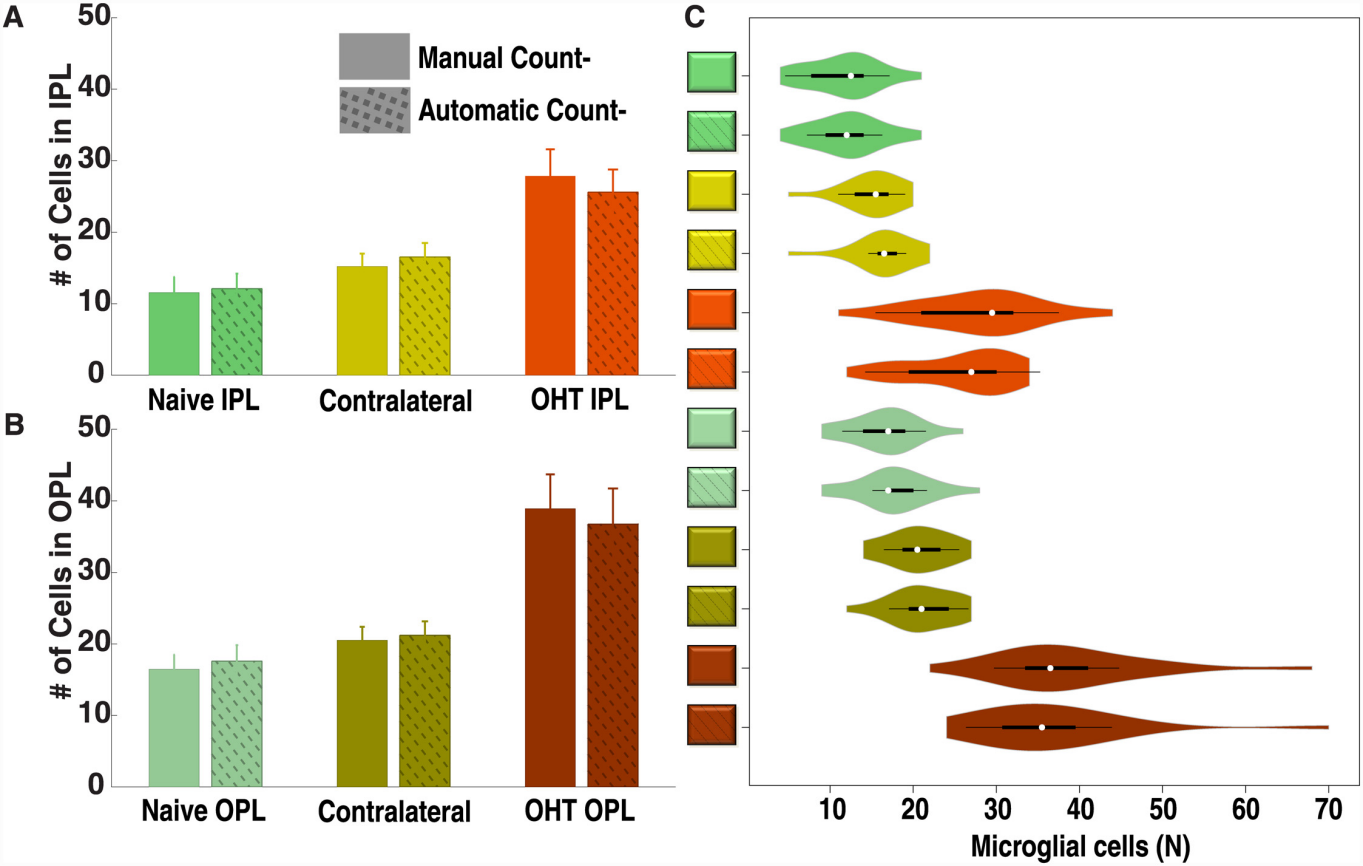
Microglial cell distribution for the automatic and manual methods. Mean±SD are shown for the three study groups, both for the manual and the automatic methods in the IPL (A) and OPL (B). (C) Distribution of the glial cell populations for the automatic and manual methods. In this plot, white circles show the medians, box limits indicate the 25th and 75th percentiles, whiskers extend 1.5 times the interquartile range from the 25th and 75th percentiles, and polygons represent density estimates of data and extend to extreme values. (IPL: inner plexiform layer; OHT: ocular hypertension; OPL: outer plexiform layer). *Used with permission from Barrow Neurological Institute*, *Phoenix*, *Arizona*.

The means and standard deviations of the samples were computed both for the manual and the automatic methods for the IPL (Fig 7A) and for the OPL (Fig 7B). A box plot [36] comparing both methods is shown in Fig 7C. Ideally each pair of figures, manual and automatic ones, should be identical.

Wilcoxon signed rank tests were performed to compare the manual and automatic methods and no statistically significant differences were found.

After validating the algorithm, it was applied to the whole set of fields recorded (Z projections) in the IPL and the OPL. The automatic method detected the number and position of cells in the IPL and OPL. The percentage of the area of the retina occupied by Iba-1+ cells of the NFL-GCL was also analyzed automatically with the threshold algorithm developed.

**Fig 8:**
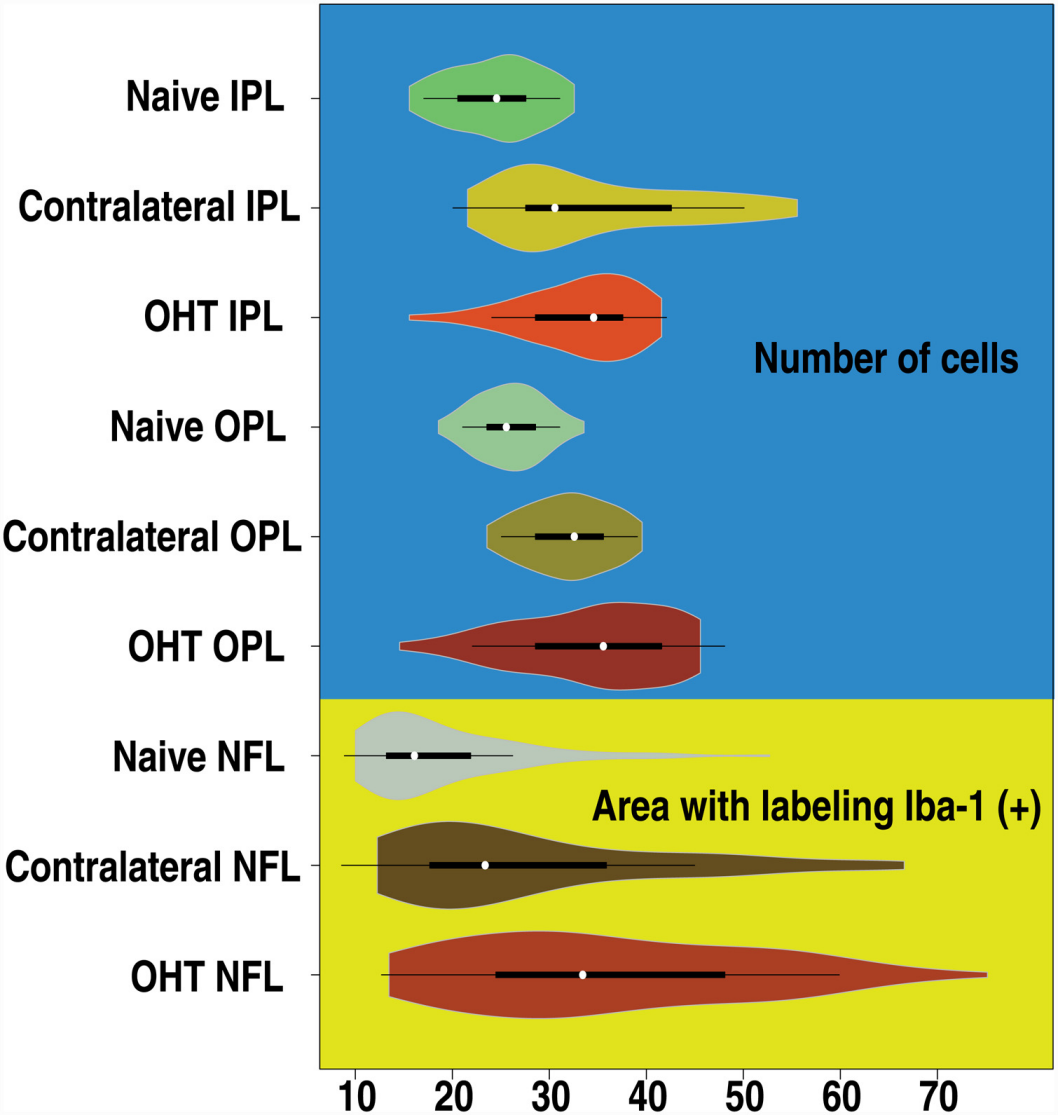
Microglial cell activation for the whole set of images measured with the automatic method (n = 1215) for the different groups of the study. White circles show the medians; box limits indicate the 25th and 75th percentiles; whiskers extend 1.5 times the interquartile range from the 25th and 75th percentiles; polygons represent density estimates of data and extend to extreme values. Values on the blue background refer to the number of microglial cells found in that layer; those on the yellow background represent the percentage of the area labeled with a reaction to Iba-1 (+). The extreme values (top 10 and bottom 10 results) were eliminated from each group. The number of samples for the each group after this elimination was 121 naïve, 116 contralateral, and 108 OHT eyes. (IPL: inner plexiform layer; NFL: nerve fiber layer; OHT: ocular hypertension; OPL: outer plexiform layer). *Used with permission from Barrow Neurological Institute*, *Phoenix*, *Arizona*.

Fig 8 shows the median, distribution, and 25th and 75th percentiles for IPL, OPL, and NFL-GCL. The 10 highest and lowest values in each subgroup were discarded. The number of samples for each group after this elimination was as follows: 121 naïve, 116 contralateral, and 108 OHT eyes.

## Discussion

We have described a new, powerful, automated, image analysis method developed with MATLAB that is capable of detecting individual immunolabeled microglial cells and immunolabeled regions in whole-mount mouse retinas using a threshold strategy.

The proliferation of microglial cells is a hallmark of the presence of potentially damaging insults or diseases in the nervous system, including the retina. Microglial counting has been used widely in neuroscience to reveal the presence of associated ongoing gliotic processes. Microglial proliferation is typically found in human glaucoma and in different glaucomatous experimental models [15–22,24,37–39], and it has also been observed with other types of retinal damage [37,38,40–46]

Quantitative assessment of microglial cells in the retina continues to be a manual process in most of the studies performed currently across the world. This manual technique is vulnerable to operator subjectivity, which may result in intra- and inter-operator variability that creates non-reproducible and imprecise experimental results. The risk is even greater if the operator is not blinded to the sample because subjective bias can occur. Despite its multiple disadvantages (e.g., it is monotonous, tedious, operator dependent, and time-consuming; slows the analysis of the samples; and introduces experimental error), the manual method is still considered the gold standard for quantitative assessments of microglial cells in the retina.

Due to the increasing applications of cell counting in the field of biology [47–50], numerous automatic software programs have been developed (e.g., Image-Pro Plus, ImageJ). Other programs have emerged in the last few years to enable detection of different cell types in the nervous system [25–27,29,51–56] including microglial cells (Indica Labs Inc.) [28,31,57], that allow image analysis based on segmentation of samples. Automatic counting methods have several benefits that improve the analysis of serial samples—they are faster, non-operator dependent (higher reproducibility), and less labor intensive than manual strategies. Various methods have also been developed for automated retinal image analysis [56,58–62]. Most of them work appropriately with cells that exhibit homogeneous and rounded shapes, but are generally less robust for irregular cell shapes, such as branched microglial cells. For example the number of apoptosing retinal ganglion cells has been counted with ImageJ; however, as far as we know, there is no evidence of an accurate and automated retinal microglial cell counting method. In the eye, microglial cells are spread throughout different retinal layers. This complex organization of microglial cells could be a reason why an automatic counting method has not yet been designed for the retina.

The algorithm developed in our laboratory allows us to count Iba-1+ cells to a level of reliability that is similar to the gold standard, while minimizing cell count variability arising from inter-observer variability. The automated cell counts have a significant correlation with the gold-standard manual method, with no significant difference between methods detected. A good correlation between manual and automatic counting was shown in our study using naïve retinas, OHT retinas, and contralateral retinas. Therefore, this new technique allows us to analyze microglial cell numbers in normal and highly proliferative gliotic states.

In some retinal layers, given the complexity of the glial response under pathological insults, it is not possible to objectively quantify the number of microglial cells. In a model of OHT, Rojas et al. [24] observed in the NFL-GCL not only an increase of the number of Iba-1+ cells but also the presence of new cell types. This fact is in accordance with our results, and we undertook methods to correct the identification given the degree of complexity and overlapping cells. Because of this, and bearing in mind that the increased expression of certain proteins, such as Iba-1, is a marker of an ongoing gliotic process, another approach to the analysis of glial reactivation involves analyzing tissue in an immunolabeled area rather than cell counting, with the positively immunolabeled area being an indirect measure of the glial reactivation. With the approach taken for this layer, other cells, i.e. perivascular macrophages that are Iba-1+ cells, can be included in the quantification of the retinal area labeled with Iba-1. Several software tools are already associated with fluorescence microscopes that allow investigators to adequately separate immunolabeled areas from the background in the images. In our study, perform thresholding and cell counting using different software slowed the analysis of the samples and also increased the possibility of confusion when putting the data back together. Therefore, we developed an extra semiautomatic tool for this algorithm to analyze the total area occupied by the Iba-1+ immunostaining in the image. Although this strategy was employed for the evaluation of the NFL-GCL, it could be applied to other retinal layers and also to analyze the increase in immunoreactivity of other proteins in microglia or other cell types.

The algorithm developed in our laboratory can operate not only in 2-dimensions but also with cells located in depth on the Z axis, using projections representing a 3-dimensional volume, which is a great advantage because the distribution of microglial cells in the retina is 3 dimensional. Each quantitative analysis takes no more than 1 second per image, which provides an advantage for analyzing a large dataset of numerous imaging samples, which is a normal routine in laboratories worldwide. The time-related benefits provided by the automatic method in comparison with the manual gold standard are substantial because a manual analysis of the mouse retina may take several days for each animal.

The interactive graphic interface we developed provides a direct visualization of the process, which is used to confirm precision, and interactive windows that allow changes in some analytic parameters, such as cell distance and image threshold. This interface makes this method extremely easy to use, which allows the user to analyze images more comfortably and successfully without requiring computation knowledge. Also, the summary of the measurements is recorded in XML format, facilitating access and analysis of results, by using Microsoft Excel for example.

Although some commercial software (e.g., Indica Labs Inc.) is already available for quantitative analysis of microglial cells, this software is expensive; in contrast, the algorithm we developed can be economically applied in retinal research groups working on microglial cells.

This algorithm has been developed taking into account the retinal microglial distribution; however, some parameters within the algorithm, such as microglial cell-cell distance or threshold, can be modified in the interactive interface developed for its application in other tissues, or with insult conditions different from glaucoma. Our method is of interest for researchers using whole-mount retinas as an in vivo experimental model; however, it is not restricted to the analysis of microglial cells. Other cells with branched morphology, similar to microglia, such as dendritic cells, could be identified and counted by this algorithm. The development of this method with MATLAB will be fundamental in future studies that try to establish a quantitative analysis for resting versus activated microglial cells in response to different scenarios known to induce microglial reactivity.

**Fig 9:**
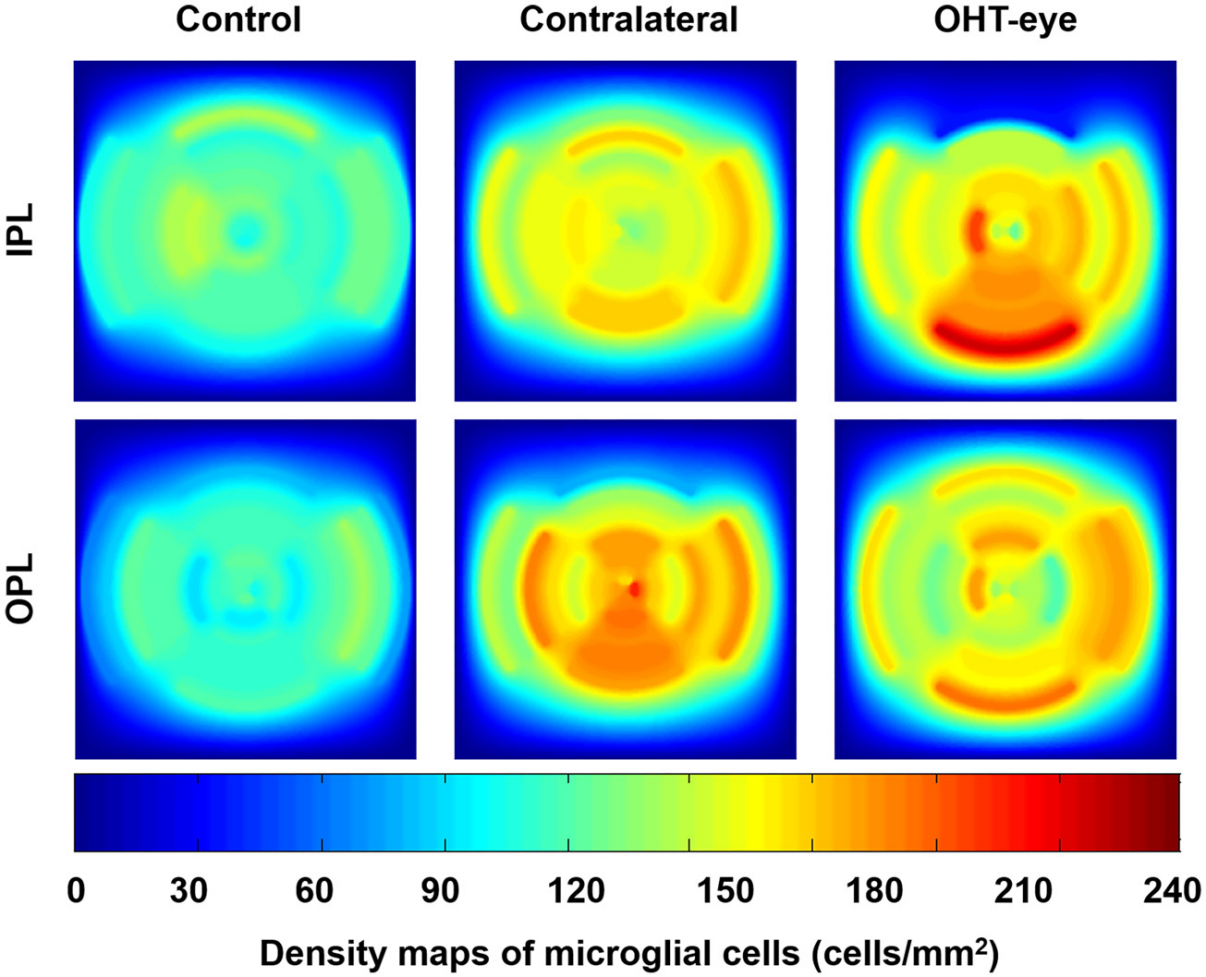
Density maps of the microglial distribution in the retina. Average density maps obtained by interpolation of the original data (upper row IPL, bottom row OPL); columns represent the 3 study groups: naïve, contralateral, and OHT eyes. (IPL: inner plexiform layer; OHT: ocular hypertension; OPL: outer plexiform layer). *Used with permission from Barrow Neurological Institute*, *Phoenix*, *Arizona*.

Because the algorithm records the position of the cells, the possibility of generating density maps of the cell populations in the retina is open (Fig 9).

The hotspot of microglia in the inferior retina shown in Fig 9 could be related to the pattern of surviving retinal ganglion cells described in the experimental model used in a previous study [32]. In this model, 2 weeks after laser-induced OHT, the time point at which our retinas were studied, the remaining ganglion cells were located in the inferior retina, the area that matches with our hotspot. Whether or not the microglia in this area are exerting neuroprotection deserves further investigation.

So far in our laboratory, samples are examined as shown in Fig 1A. A more exhaustive scan of the retina is necessary to perfect the implementation of a density map recording not only the vertical and horizontal meridians but also the areas in the oblique directions.

## Conclusions

A new, reliable, and rapid algorithmic was developed under MATLAB to obtain the number and position of microglial cells in control and other proliferative conditions (e.g., glaucoma) in the IPL and the OPL. The algorithm also offers the possibility of identifying the immunolabeled area on the NFL-GCL. Using this automated segmentation method in parallel with immunohistochemistry staining the process of counting a set of ~6,000 images can be dramatically reduced from weeks (manual counting) to a little less than 1 hour (conventional personal computer); the position of the cells is recorded, which allows new ways to analyze the samples; and differences between the results obtained with the automatic method and the direct human inspection were not statistically significantly different. The number of cells present in the sample was not an obstacle for the program to run properly in the IPL, the OPL, and the NFL-GCL. The program will be made freely available by the authors to anyone who contacts them. This algorithm requires MATLAB installed on the computer on which the user run the program.

## Abbreviations

GCL: ganglion cell layer
IPL: inner plexiform layer
NFL: nerve fiber layer
OHT: ocular hypertension
OPL: outer plexiform layer
